# Ferritin-Based Biomarkers for Prediction of 28-Day Mortality in Septic Shock: A Prospective, Single-Centre, Observational Study From South India

**DOI:** 10.7759/cureus.115711

**Published:** 2026-09-03

**Authors:** Kaveri Bobbala, Ann Mary, Sanjeev M George, Renoy A Henry, Devika Saju

**Affiliations:** 1 Internal Medicine, Amrita Institute of Medical Sciences, Kochi, IND; 2 Biostatistics, Amrita Institute of Medical Sciences, Kochi, IND

**Keywords:** ferritin-albumin ratio, ferritin index, hyperferritinemia, mortality prediction, septic shock

## Abstract

Introduction

Septic shock is a life-threatening condition with high mortality despite advances in critical care. Ferritin, an acute-phase reactant involved in iron metabolism and inflammation, along with its derived biomarkers, has emerged as a potential biomarker of disease severity in sepsis.

Methods

This prospective observational study was conducted in an Intensive Care Unit in a Tertiary Care Hospital from December 2024 to October 2025. Adult patients meeting Sepsis-3 criteria for septic shock were included. Serum ferritin was measured on Day 1 and Day 3. Ferritin index, Delta ferritin and Ferritin-albumin ratio were calculated. Receiver operating characteristic (ROC) curve analysis was performed to evaluate the discriminatory performance of serum ferritin, Ferritin Index (FI), delta ferritin (ΔFerritin), and ferritin-albumin ratio (FAR). Optimal cutoffs were determined using the ROC curves, and pairwise comparisons of the areas under the curves (AUCs) were performed using DeLong’s test. Univariate and multivariable logistic regression analyses were performed to identify predictors of 28-day mortality.

Results

A total of 79 patients were included, with a 28-day mortality rate of 64.6%. All ferritin-based biomarkers were significantly associated with 28-day mortality. Among these, the ferritin-albumin ratio demonstrated the best predictive performance (AUC: 0.849), with a sensitivity of 82.5% and specificity of 81.8% at a cutoff value of ≥239. Day 1 ferritin also showed excellent predictive ability (AUC: 0.840), with 94.1% sensitivity and 60.7% specificity at a cutoff of ≥574 µg/L. The ferritin index showed good discrimination (AUC: 0.795; sensitivity: 72.6%; specificity: 78.6% at ≥5.45), while delta ferritin demonstrated acceptable predictive performance (AUC: 0.737; sensitivity: 96.0%; specificity: 67.5% at ≥305 µg/L). DeLong pairwise comparison demonstrated that FAR was significantly superior to Ferritin Index, but its performance did not differ significantly from Day 1 serum ferritin or delta ferritin. On univariate analysis, Day 1 ferritin, Ferritin Index, FAR, SOFA, qSOFA, and lactate were associated with mortality; however, SOFA remained the only independent predictor in multivariable analysis.

Conclusion

Ferritin-derived biomarkers serve as valuable prognostic tools for risk stratification in septic shock. While the ferritin-albumin ratio demonstrated high diagnostic accuracy, the ferritin index and delta ferritin also show clinical utility. These inexpensive and widely available markers may aid early risk stratification in critically ill patients, though their prognostic value is closely aligned with baseline clinical severity scores.

## Introduction

Sepsis is a life-threatening syndrome characterized by organ dysfunction resulting from a dysregulated host response to infection [1]. Septic shock, the most severe manifestation of sepsis, is defined by persistent hypotension requiring vasopressor support despite adequate fluid resuscitation, accompanied by metabolic derangements such as elevated serum lactate levels [2]. Although substantial advances in antimicrobial therapy, hemodynamic monitoring, and critical care have improved patient management, septic shock continues to be associated with considerable morbidity and mortality worldwide [3].

The pathogenesis of sepsis is multifactorial and involves a complex interplay of excessive inflammatory responses, immune dysregulation, endothelial injury, microcirculatory dysfunction, coagulation abnormalities, and metabolic disturbances [4,5]. These interconnected mechanisms contribute to progressive tissue hypoperfusion, multiple organ dysfunction, and an increased risk of death.

Ferritin, an intracellular iron-storage protein, also serves as an important acute-phase reactant during systemic inflammatory states [6]. In sepsis, pro-inflammatory cytokines stimulate ferritin synthesis and release, resulting in elevated circulating ferritin concentrations [7]. Beyond reflecting iron metabolism, hyperferritinemia has been implicated in immune dysregulation, macrophage activation, oxidative stress, and the amplification of inflammatory pathways in critically ill patients [8].

Nevertheless, the prognostic utility of serum ferritin alone is limited because its concentration may be influenced by a variety of physiological and pathological conditions, including chronic liver disease, malignancy, chronic inflammatory disorders, and other non-infectious inflammatory states [9]. To address these limitations, several ferritin-derived biomarkers, including the ferritin index, delta ferritin, and ferritin-albumin ratio, have been proposed to better characterize the inflammatory milieu and enhance risk stratification in critically ill patients.

Recent evidence suggests that composite biomarkers integrating inflammatory markers with physiological or biochemical parameters may provide superior prognostic performance compared with individual biomarkers alone [10]. In this context, the present study aimed to evaluate the prognostic value of ferritin-based biomarkers, including the Ferritin index (FI), Delta ferritin (ΔFerritin), and the Ferritin-albumin ratio (FAR) for predicting 28-day mortality among patients with septic shock. We hypothesized that ferritin-derived biomarkers, particularly composite biomarkers, would demonstrate good discriminatory ability for predicting 28-day mortality in patients with septic shock.

## Materials and methods

Study design and setting

This prospective observational study was conducted in the intensive care unit of a tertiary care hospital in South India between December 2024 and October 2025. The study was approved by the Institutional Ethics Committee, and informed consent was obtained from all participants prior to enrollment.

The sample size was calculated based on a pilot study involving 12 patients, which demonstrated a sensitivity of 50% for the ferritin index in predicting 28-day mortality. Assuming a 95% confidence level and a 20% allowable error, the minimum required number of mortality events was estimated to be 24. Considering an anticipated mortality incidence of 33.3%, the overall minimum sample size was calculated to be 72 patients.

Patient selection

A total of 79 patients with sepsis and septic shock who met the eligibility criteria were included in the study. Patients admitted to the intensive care unit (ICU) with septic shock were screened for eligibility. Septic shock was defined according to the Sepsis-3 criteria as suspected or documented infection associated with an increase in the Sequential Organ Failure Assessment (SOFA) score of ≥2, requiring vasopressor therapy to maintain a mean arterial pressure (MAP) of ≥65 mmHg and having a serum lactate level >2 mmol/L despite adequate fluid resuscitation. Patients meeting these criteria were included in the study. Patients with conditions known to significantly alter ferritin levels independent of sepsis, which included hemochromatosis, severe iron deficiency anemia, recent massive blood transfusion, hemophagocytic lymphohistiocytosis (HLH), and adult-onset Still's disease (AOSD), were excluded from the study. The primary source of infection was determined by the treating physicians based on a combination of clinical evaluation, microbiological culture results, and radiological findings. Respiratory, bloodstream, urinary tract, gastrointestinal, and other infections were classified according to the predominant site of infection documented at the time of ICU admission.

Data collection

Data were collected prospectively using a standardized case record form. Baseline demographic characteristics, comorbidities, clinical presentation, source of infection, laboratory investigations, Sequential Organ Failure Assessment (SOFA) score, vasopressor requirement, and serum lactate levels were recorded at the time of ICU admission. Complete baseline demographic, clinical, and laboratory data were successfully obtained for all 79 enrolled patients with no missing baseline variables. All patients completed the 28-day follow-up period with no loss to follow-up. Serum ferritin level was measured on day 1 (within 24 hours of ICU admission) and repeated on day 3. Serum albumin levels were measured on day 1. The ferritin index was calculated as the ratio of the observed serum ferritin level to the sex-specific upper limit of normal (300 µg/L for males and 150 µg/L for females). Delta ferritin was calculated as the difference between serum ferritin levels on day 3 and day 1 among patients who survived to day 3 (14 patients were excluded due to death within the first 48 hours of admission). The ferritin-albumin ratio was calculated by dividing the serum ferritin concentration (µg/L) by the serum albumin concentration (g/dL). Patients were followed for 28 days after ICU admission to determine all-cause mortality.

The following derived biomarkers were calculated:

Ferritin Index

\[ \text{Ferritin Index} = \frac{\text{Observed Ferritin }(\mu\text{g/L})} {\text{Sex-specific ULN }(\mu\text{g/L})} \]

The upper limit of normal (ULN) for serum ferritin was defined as 300 µg/L for males and 150 µg/L for females, and these values were used to calculate the Ferritin Index.

Delta Ferritin

\[ \Delta\text{Ferritin} = \text{Ferritin}_{\text{Day 3}}(\mu\text{g/L}) - \text{Ferritin}_{\text{Day 1}}(\mu\text{g/L}) \]

Ferritin-Albumin Ratio

\[ \text{Ferritin-Albumin Ratio (FAR)} = \frac{\text{Ferritin }(\mu\text{g/L})} {\text{Albumin }(\text{g/dL})} \]

Laboratory methods

Blood samples were collected within 24 hours of ICU admission (day 1), and serum ferritin estimation was repeated on day 3. Serum ferritin was measured using an electrochemiluminescence immunoassay (ECLIA) on the Roche Cobas 8000 analyzer series (Roche Diagnostics, Germany). Serum albumin was measured on day 1 using a colorimetric assay on the same analyzer, in accordance with the manufacturer's instructions. All laboratory analyses were performed in the hospital's central clinical laboratory following routine internal and external quality control procedures.

Outcome measured

The primary outcome was 28-day all-cause mortality, defined as death from any cause occurring within 28 days of ICU admission.

Statistical analysis

Statistical analysis was performed using IBM SPSS Statistics for Windows, Version 20.0 (IBM Corp., Armonk, NY, USA). Receiver operating characteristic (ROC) curve analysis was used to evaluate the discriminatory performance of the ferritin index, serum ferritin on day 1, delta ferritin, and the ferritin-albumin ratio for predicting 28-day mortality and to determine their optimal cutoff values. Associations between categorical variables and 28-day mortality were analyzed using the Pearson chi-square test or Fisher’s exact test, as appropriate. ROC curves were compared using the DeLong method to assess differences in AUCs. Univariate and multivariate logistic regression analyses were performed to identify factors independently associated with 28-day mortality, with results reported as odds ratios (ORs) with 95% confidence intervals (CIs). A two-sided P value of <0.05 was considered statistically significant.

## Results

Demographic characteristics

A total of 79 patients with septic shock were included in the study. The mean age of the study population was 64.9 ± 12.2 years (range: 29-89 years). There were 48 (60.8%) male patients and 31 (39.2%) female patients.

Comorbidity profile

Pre-existing comorbidities were common among the study population. Diabetes mellitus was the most frequently observed comorbidity, affecting 46 (58.2%) patients, followed by hypertension in 28 (35.4%), chronic kidney disease in 6 (7.6%), coronary artery disease in 5 (6.3%), and malignancy in 5 (6.3%) patients.

Baseline comorbidities are summarized in Table 1.

**Table 1:** Comorbidity profile of the study population (n = 79)

Comorbidity	Number (n)	Percentage (%)
Diabetes Mellitus	46	58.2
Hypertension	28	35.4
Thyroid Disorders	7	8.9
Chronic Kidney Disease	6	7.6
Coronary Artery Disease	5	6.3
Dyslipidemia	5	6.3
Malignancy	5	6.3
Chronic Lung Disease	3	3.8
Cerebrovascular Disease	1	1.3
Autoimmune Disorders	2	2.5
Valvular Heart Disease	2	2.5
No Comorbidity	4	5.1

Etiological sources of sepsis

Respiratory tract infections were the most common source of sepsis, accounting for 36 (45.6%) cases. Bloodstream infections were identified in 17 (21.5%) patients, followed by urinary tract infections in 13 (16.5%) and gastrointestinal infections in 8 (10.1%) patients. Other sources of infection were observed in 5 (6.3%) patients. The distribution of the etiological sources of sepsis is presented in Table 2.

**Table 2:** Etiological sources of sepsis

Source	N (%)
Respiratory infections	36 (45.6)
Bloodstream infections	17 (21.5)
Urinary infections	13 (16.5)
Gastrointestinal infections	8 (10.1)
Other infections	5 (6.3)

Mortality outcomes

The primary outcome assessed in this study was 28-day mortality. Among the 79 patients included in the study, it was found that 51 patients (64.6%) died and 28 patients (35.4%) survived. This mortality rate reflects the severity of illness in patients presenting with septic shock. Receiver operating characteristic (ROC) analysis was performed to determine the prognostic value of ferritin-based biomarkers.

Serum ferritin (day 1) and 28-day mortality

All 79 patients had day 1 serum ferritin value. Receiver operating characteristic (ROC) curve analysis demonstrated that serum ferritin measured on ICU day 1 had good discriminatory ability for predicting 28-day mortality, with an area under the curve (AUC) of 0.840 (95% CI: 0.745-0.934; p < 0.001).

The optimal cutoff value was ≥574 µg/L, which yielded a sensitivity of 94.12% (95% CI: 83.76%-98.77%), a specificity of 60.71% (95% CI: 40.58%-78.50%), a positive predictive value of 81.36%, a negative predictive value of 85.00%, and an overall diagnostic accuracy of 82.28%. Using this cutoff, 59/79 patients (74.7%) had serum ferritin levels ≥574 µg/L, of whom 48 (81.4%) died within 28 days and 11 (18.6%) survived. In contrast, among the 20/79 patients (25.3%) with serum ferritin levels <574 µg/L, only 3 (15.0%) died, whereas 17 (85.0%) survived. Serum ferritin levels ≥574 µg/L were significantly associated with 28-day mortality (χ² = 28.744, p < 0.001).

The ROC curve and the distribution of 28-day mortality according to the optimal serum ferritin cutoff are presented in Figure 1 and Table 3, respectively.

**Figure 1:**
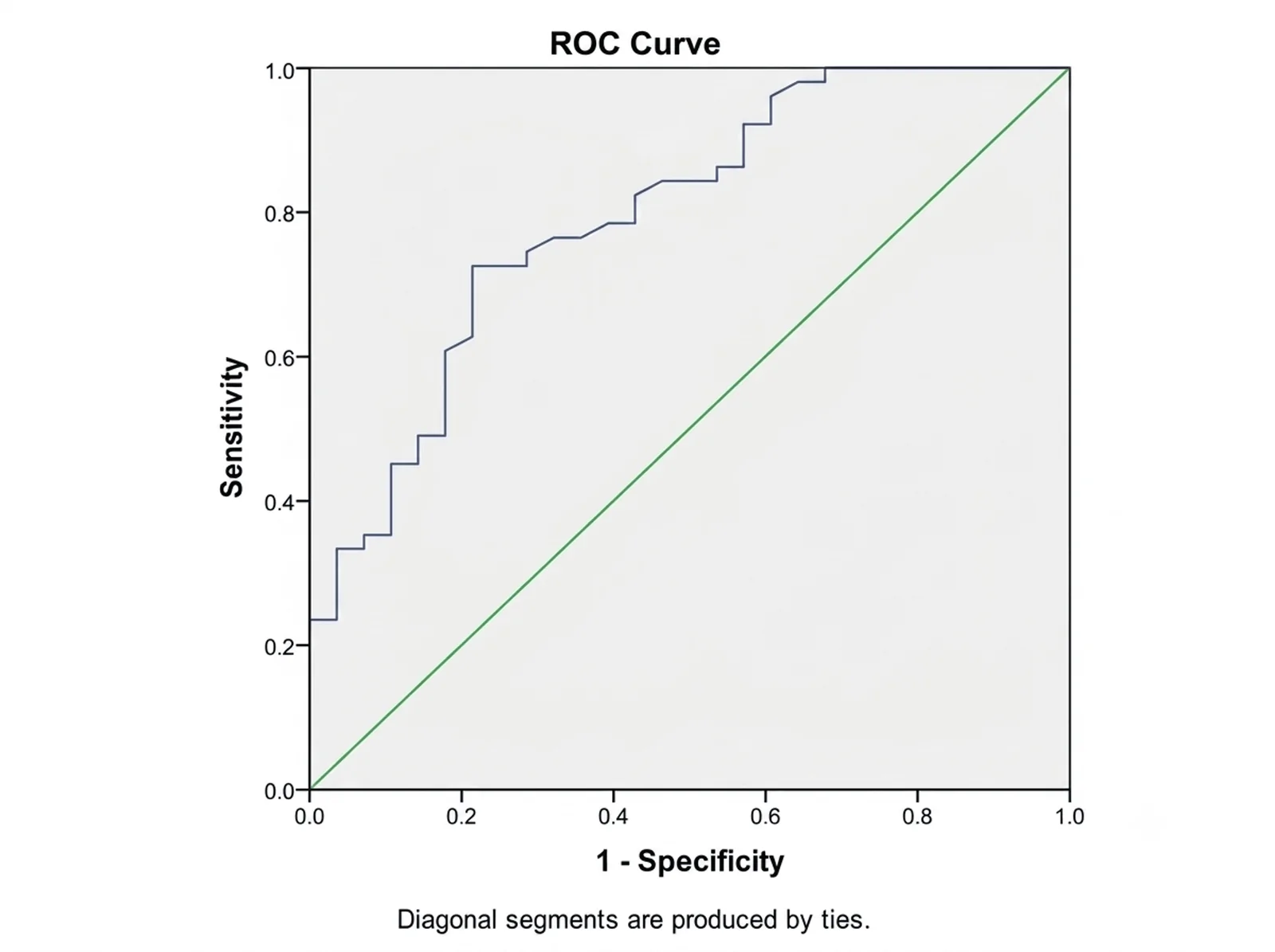
ROC curve- serum ferritin day 1

**Table 3:** Association between day 1 serum ferritin cutoff and 28-day mortality

Day 1 Serum Ferritin (µg/L)	28-Day Mortality, n (%)	Survived, n (%)	Total, n
≥574	48 (81.4)	11 (18.6)	59
<574	3 (15.0)	17 (85.0)	20
Total	51 (64.6)	28 (35.4)	79

Ferritin Index and 28-day mortality

All 79 patients had complete data and were included in the analysis. Of these, 51 (64.6%) died within 28 days, while 28 (35.4%) survived. ROC curve analysis demonstrated that the Ferritin Index had good discriminatory ability for predicting 28-day mortality, with an AUC of 0.795 (95% CI: 0.693-0.898; p < 0.001).

Using the optimal ROC-derived cutoff value of ≥5.45, 43 patients were classified as having an elevated Ferritin Index. Among these, 37 (86.0%) died within 28 days, whereas 6 (14.0%) survived. In contrast, among patients with a Ferritin Index <5.45, 14 (38.9%) died, and 22 (61.1%) survived. The association between an elevated Ferritin Index and 28-day mortality was statistically significant (Pearson χ² = 19.045, p < 0.001).

At the cutoff value of ≥5.45, the Ferritin Index demonstrated a sensitivity of 72.55% (95% CI: 58.26%-84.11%), specificity of 78.57% (95% CI: 59.05%-91.70%), positive predictive value of 86.05%, negative predictive value of 61.11%, and an overall diagnostic accuracy of 74.68%.

ROC curve and the distribution of 28-day mortality according to the optimal ferritin index cutoff are presented in Figure 2 and Table 4, respectively.

**Figure 2:**
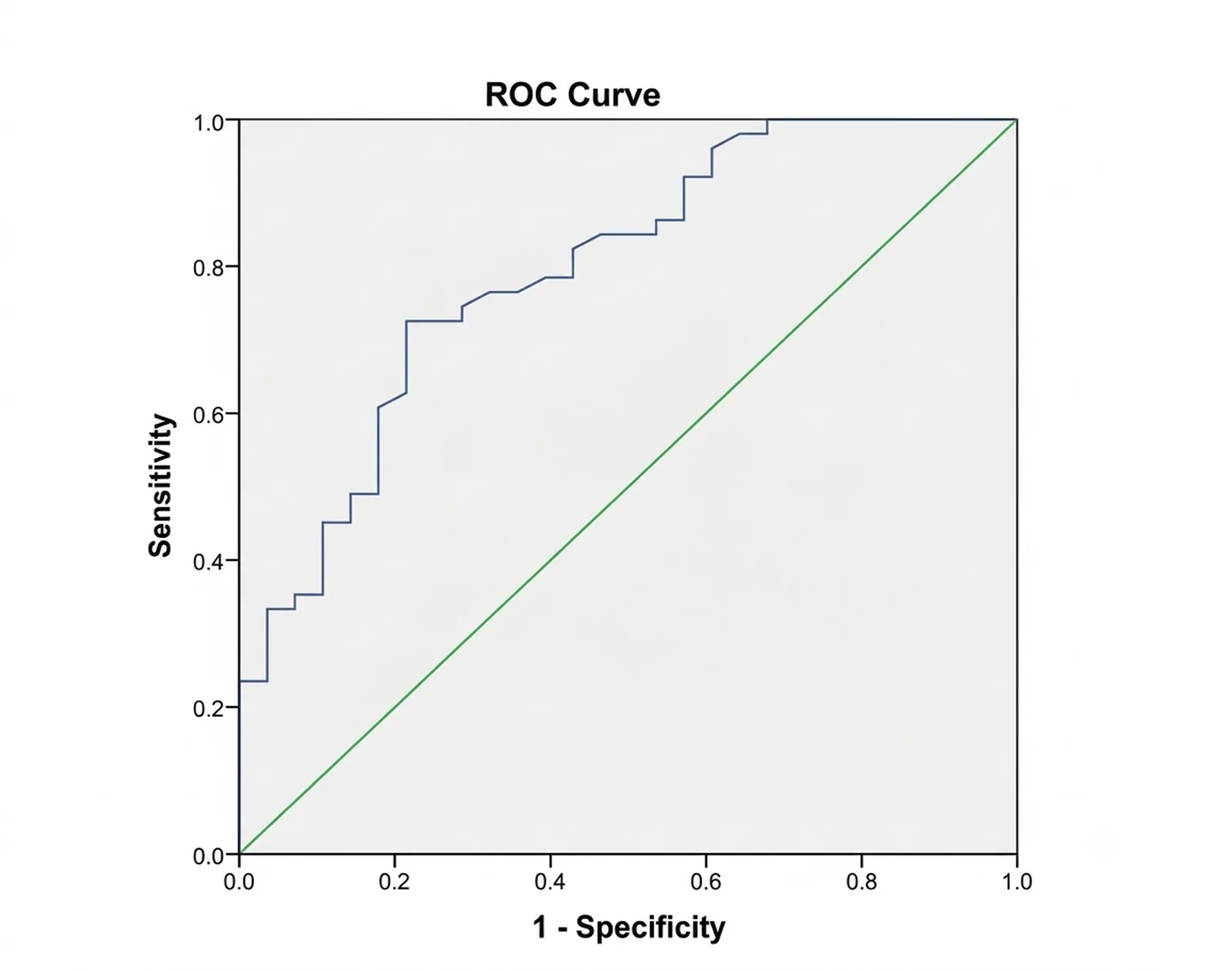
ROC curve- ferritin index

**Table 4:** Association between ferritin index cutoff and 28-day mortality

Ferritin Index	28-Day Mortality, n (%)	Survived, n (%)	Total, n
≥5.45	37 (86.0)	6 (14.0)	43
<5.45	14 (38.9)	22 (61.1)	36
Total	51 (64.6)	28 (35.4)	79

Delta ferritin and 28-day mortality

Delta ferritin was calculated as the difference between ferritin levels measured on day 3 and day 1. Delta ferritin analysis was performed in 65 patients with serial ferritin measurements available, excluding 14 patients who died before day 3. ROC curve analysis demonstrated acceptable predictive performance for 28-day mortality, with an AUC of 0.737 (95% CI: 0.610-0.865; p = 0.001) (Figure 3).

The optimal cutoff value was ≥305 µg/L. At this threshold, 24 of 25 patients (96.0%) died within 28 days compared with 13 of 40 patients (32.5%) with delta ferritin <305 µg/L (Table 5). Elevated delta ferritin was significantly associated with 28-day mortality (Pearson χ² = 25.299, p < 0.001). The cutoff demonstrated a sensitivity of 96.0% (95% CI: 79.65%-99.90%), specificity of 67.5% (95% CI: 50.87%-81.43%), positive predictive value of 64.9%, negative predictive value of 96.4%, and an overall diagnostic accuracy of 78.5%.

ROC curve and the distribution of 28-day mortality according to the optimal Delta Ferritin cutoff are presented in Figure 3 and Table 5, respectively.

**Figure 3:**
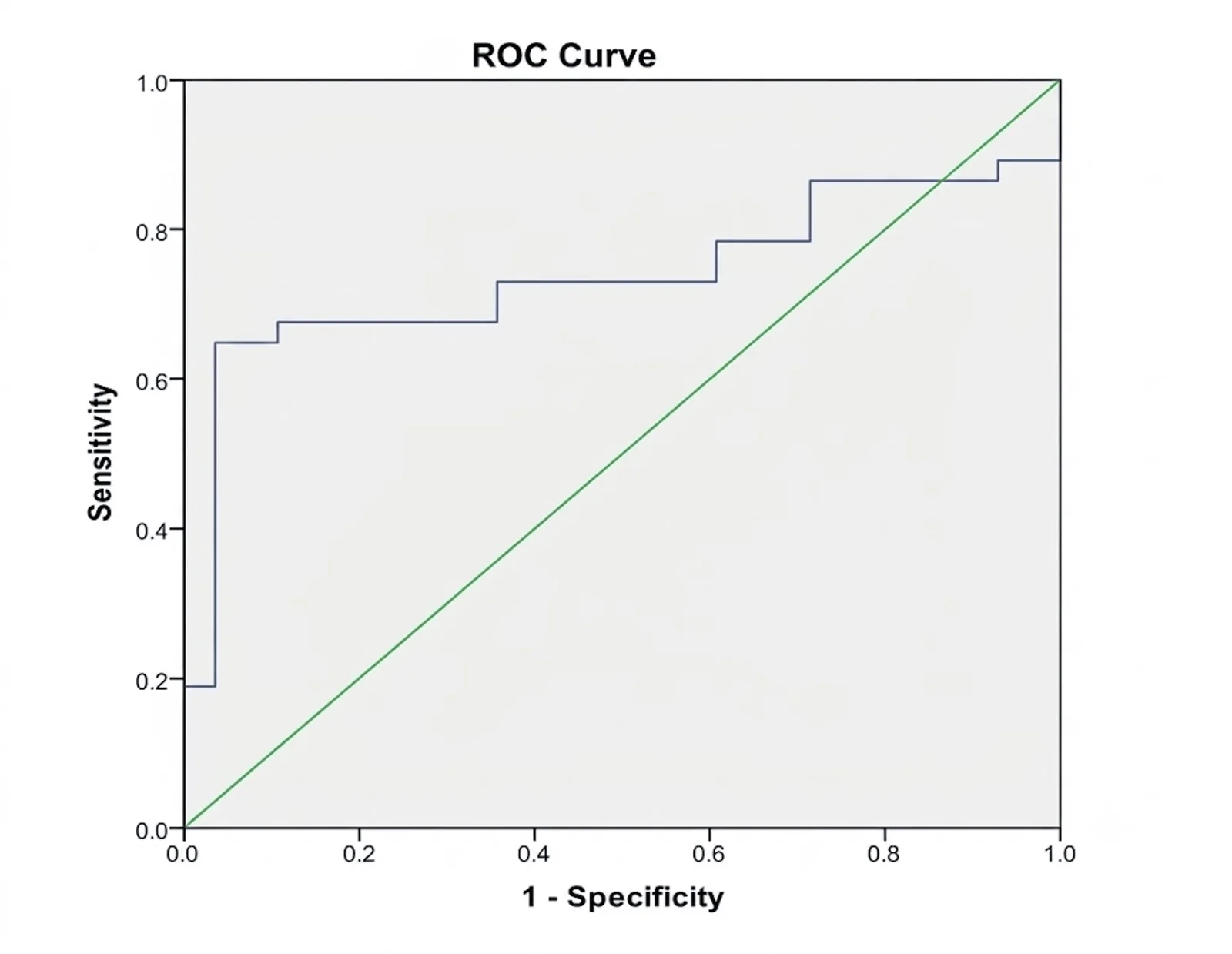
ROC curve- delta ferritin

**Table 5:** Association between delta ferritin cutoff and 28-day mortality

Delta Ferritin (µg/L)	28-Day Mortality, n (%)	Survived, n (%)	Total, n
≥305	24 (96.0)	1 (4.0)	25
<305	13 (32.5)	27 (67.5)	40
Total	37 (56.9)	28 (43.1)	65

Ferritin-albumin ratio and 28-day mortality

All 79 patients had complete ferritin-albumin ratio data and were included in the analysis. The ferritin-albumin ratio demonstrated excellent predictive performance for 28-day mortality, with an AUC of 0.849 (95% CI: 0.759-0.940; p < 0.001) (Figure 4). The optimal cutoff value was ≥239. At this threshold, 47 of 57 patients (82.5%) died within 28 days compared with 4 of 22 patients (18.2%) with a ratio <239 (Table 6). The association between an elevated ferritin-albumin ratio and 28-day mortality was statistically significant (Pearson χ² = 28.660, p < 0.001). The ferritin-albumin ratio demonstrated a sensitivity of 82.5%, specificity of 81.8%, positive predictive value of 92.2%, negative predictive value of 64.3%, and an overall diagnostic accuracy of 82.3%.

ROC curve and the distribution of 28-day mortality according to the optimal ferritin-albumin ratio cutoff are presented in Figure 4 and Table 6, respectively.

**Figure 4:**
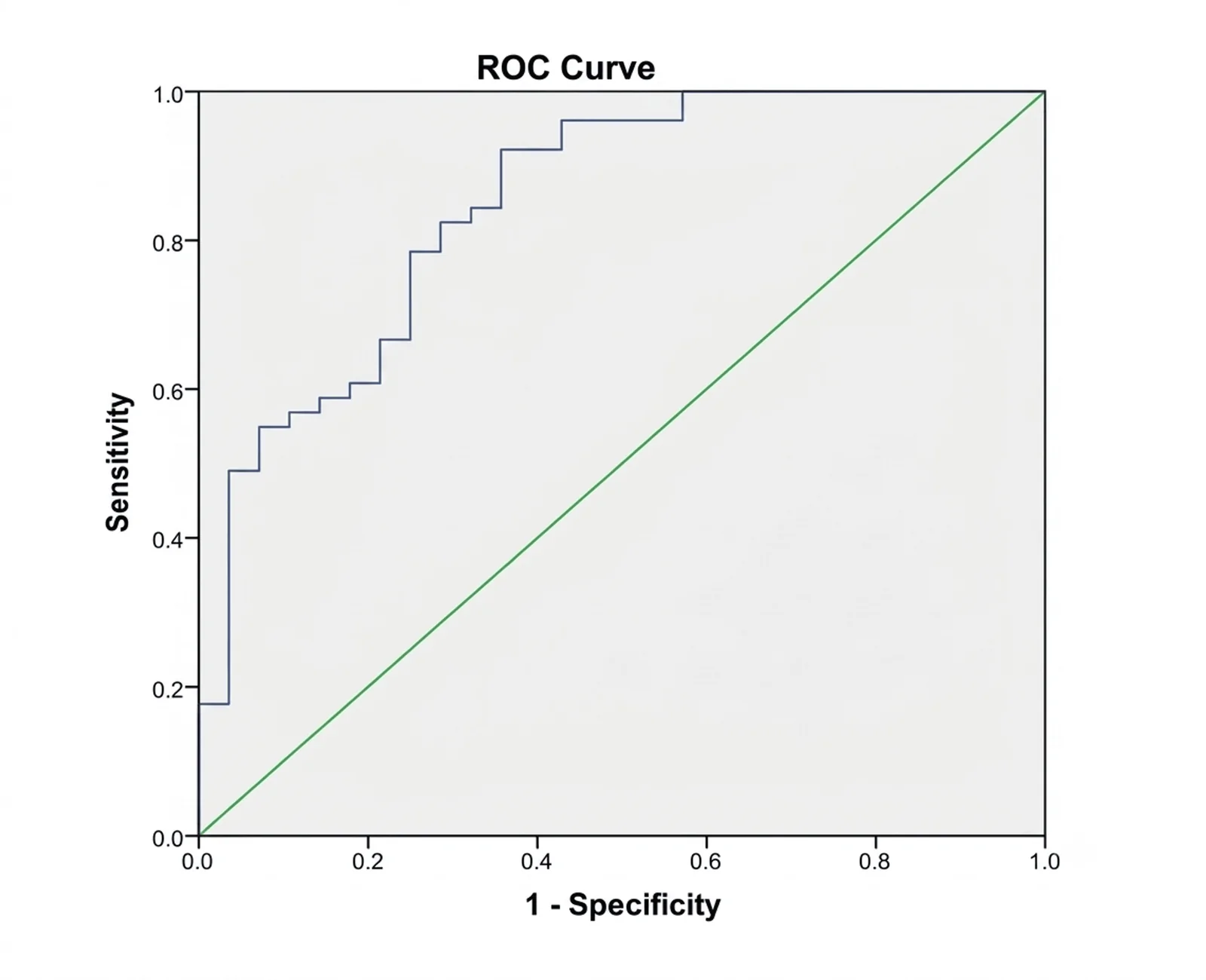
ROC curve- ferritin/albumin ratio

**Table 6:** Association between ferritin–albumin ratio cutoff and 28-day mortality

Ferritin–Albumin Ratio	28-Day Mortality, n (%)	Survived, n (%)	Total (n)
≥239	47 (82.5)	10 (17.5)	57
<239	4 (18.2)	18 (81.8)	22
Total	51 (64.6)	28 (35.4)	79

The diagnostic performance of the evaluated ferritin-based biomarkers for predicting 28-day mortality is summarized in Table 7.

**Table 7:** Diagnostic performance of ferritin biomarkers

Marker	AUC	Sensitivity (%)	Specificity (%)	PPV (%)	NPV (%)	Accuracy (%)
Ferritin Index	0.795	72.5	78.57	86.05	61.11	74.7
Serum Ferritin Day 1	0.840	94.1	60.71	81.36	85.00	82
Delta Ferritin	0.737	96.00	67.50	64.86	96.43	78.46
Ferritin / Albumin Ratio	0.849	82.46	81.82	92.46	64	82

DeLong comparison of ROC curves

ROC analysis was done which showed that the ferritin-albumin ratio (FAR) had the highest discriminatory ability for 28-day mortality (AUC = 0.849, 95% CI: 0.759-0.940; p < 0.001), followed by day 1 serum ferritin (AUC = 0.840, 95% CI: 0.744-0.935), ferritin index (AUC = 0.795, 95% CI: 0.692-0.898), and delta ferritin (AUC = 0.737, 95% CI: 0.609-0.866). Pairwise comparison using DeLong’s test, as summarised in Table 8, demonstrated that FAR had a significantly higher AUC than the ferritin index (AUC difference = 0.0543; 95% CI: 0.0087-0.0998; p = 0.020), whereas its differences from day 1 serum ferritin and delta ferritin were not statistically significant (p = 0.153 and p = 0.196, respectively).

**Table 8:** Pairwise comparison of ROC curves using DeLong's test for prediction of 28 day mortality

Biomarker comparison	AUC 1	AUC 2	Difference in AUC	95% CI for difference	z	p-value
FAR vs. Day 1 serum ferritin	0.849	0.84	0.0098	−0.0036 to 0.0232	1.43	0.153
FAR vs. Ferritin Index	0.849	0.795	0.0543	0.0087 to 0.0998	2.33	0.02
FAR vs. Delta ferritin	0.849	0.737	0.112	−0.0524 to 0.2550	1.29	0.196

Comparison of clinical and laboratory variables according to 28-day mortality

The 79 patients were analyzed and stratified according to 28-day mortality into survivors (Alive, n = 28) and non-survivors (Dead, n = 51). Non-survivors exhibited significantly higher baseline inflammatory and organ dysfunction markers compared to survivors (Table 9).

**Table 9:** Comparison of clinical and laboratory variables according to 28-day mortality

Variable	Alive (n = 28)	Dead (n = 51)	p-value
Day 1 serum ferritin (µg/L)	508.5 (264–1577.25)	3590 (1131–8422)	<0.001
Ferritin Index	2.46 (0.7–5)	9.3 (3.8–34)	<0.001
Delta Ferritin	-1 (-263.75–105)	1096 (-48.5–6728)*	0.001
Ferritin–albumin ratio	162.4 (99.15–586.5)	1121 (473–2882)	<0.001
SOFA score	6 (5–7)	9 (8–12)	<0.001
qSOFA score	1 (1–2)	2 (2–3)	<0.001
Lactate (mmol/L)	1.9 (1.12–2.3)	3.7 (1.7–6.7)	<0.001
Length of ICU stay (days)	4.5 (3–6)	7 (3–12)	0.154
Procalcitonin (ng/mL)	7.05 (1.51–37.25)	7 (2.3–21)	0.693
CRP (mg/L)	134 (96.75–280.5)	174 (81–261)	0.935

The median day 1 serum ferritin was significantly higher in non-survivors than in survivors (3,590 [IQR: 1,131-8,422] µg/L vs. 508.5 [IQR: 264-1,577.25] µg/L; p < 0.001). Similarly, non-survivors demonstrated a higher Ferritin Index (9.3 [IQR: 3.8-34] vs. 2.46 [IQR: 0.7-5]; p < 0.001), greater Delta Ferritin (1,096 [IQR: -48.5-6,728] vs. -1 [IQR: -263.75-105]; p = 0.001), and an elevated ferritin-albumin ratio (1,121 [IQR: 473-2,882] vs. 162.4 [IQR: 99.15-586.5]; p < 0.001).

Severity-of-illness scores and metabolic markers were also markedly worse in the non-survivor cohort:

· SOFA score: 9 (IQR: 8-12) vs. 6 (IQR: 5-7); p < 0.001

· qSOFA score: 2 (IQR: 2-3) vs. 1 (IQR: 1-2); p < 0.001

· Serum lactate: 3.7 (IQR: 1.7-6.7) mmol/L vs. 1.9 (IQR: 1.12-2.3) mmol/L; p < 0.001

No statistically significant differences were observed between survivors and non-survivors regarding ICU length of stay (4.5 [IQR: 3-6] vs. 7 [IQR: 3-12] days; p = 0.154), procalcitonin (7.05 [IQR: 1.51-37.25] vs. 7 [IQR: 2.3-21] ng/mL; p = 0.693), or C-reactive protein (134 [IQR: 96.75-280.5] vs. 174 [IQR: 81-261] mg/L; p = 0.935).

Logistic regression analysis for predictors of mortality

Univariate and multivariable logistic regression analyses were conducted to determine independent predictors of 28-day mortality (Table 10).

On univariate analysis, significant associations with 28-day mortality were identified for:

Day 1 serum ferritin: OR = 1.0003 (95% CI: 1.000-1.001; p = 0.014)

Ferritin Index: OR = 1.076 (95% CI: 1.014-1.143; p = 0.016)

Ferritin-albumin ratio: OR = 1.001 (95% CI: 1.000-1.001; p = 0.014)

SOFA score: OR = 1.815 (95% CI: 1.363-2.416; p < 0.001)

qSOFA: OR = 3.504 (95% CI: 1.687-7.279; p = 0.001)

Lactate: OR = 1.759 (95% CI: 1.240-2.494; p = 0.002)

Delta ferritin did not demonstrate a statistically significant association with mortality on univariate modeling (OR = 1.000, 95% CI: 1.000-1.000; p = 0.227).

**Table 10:** Univariate logistic regression analysis to determine independent predictors of 28-day mortality

Variable	Univariate OR (95% CI)	p-value
Day 1 serum ferritin	1.0003 (1.000–1.001)	0.014
Ferritin Index	1.076 (1.014–1.143)	0.016
Delta Ferritin	1.000 (1.000–1.000)	0.227
Ferritin–albumin ratio	1.001 (1.000–1.001)	0.014
SOFA score	1.815 (1.363–2.416)	<0.001
qSOFA	3.504 (1.687–7.279)	0.001
Lactate	1.759 (1.240–2.494)	0.002

Variables selected for multivariable modeling included the Ferritin Index, SOFA score, and qSOFA. In the adjusted model, only the SOFA score remained an independent predictor of 28-day mortality (adjusted OR [aOR] = 1.781, 95% CI: 1.292-2.455; p < 0.001). After adjusting for severity scores, the Ferritin Index (aOR = 1.045, 95% CI: 0.990-1.104; p = 0.108) and qSOFA (aOR = 1.902, 95% CI: 0.977-3.704; p = 0.059) were not independently associated with 28-day mortality.

## Discussion

Septic shock remains a major cause of mortality among critically ill patients despite advances in antimicrobial therapy and supportive care [3]. This study evaluated the prognostic performance of ferritin-based biomarkers for predicting 28-day mortality in patients with septic shock.

Ferritin is increasingly recognized as a biomarker of systemic inflammation, reflecting cytokine-mediated immune activation and dysregulated iron metabolism during sepsis [6,7]. Previous studies have demonstrated that hyperferritinemia is associated with greater disease severity and increased mortality in septic patients [11,12]. Although serum ferritin measured on ICU day 1 showed good predictive performance in the present study (AUC 0.840), its prognostic value may be influenced by underlying comorbidities and other inflammatory conditions, limiting its specificity [9].

To improve prognostic assessment, composite and dynamic ferritin-derived biomarkers were evaluated. The Ferritin index (Observed Ferritin ÷ Upper Limit of Normal) demonstrated good discrimination for 28-day mortality (AUC 0.795), supporting previous observations that ferritin-derived indices provide additional prognostic information beyond absolute ferritin values [13]. Sepsis is characterized by rapidly evolving inflammatory responses; serial biomarker measurements may better capture disease progression than a single baseline measurement [14-16]. Delta ferritin (Ferritin (Day 3) − Ferritin (Day 1)), which reflects temporal changes in ferritin concentrations, showed acceptable predictive performance (AUC 0.737).

Among all biomarkers evaluated, the ferritin-albumin ratio exhibited the highest predictive performance (AUC 0.849), with balanced sensitivity and specificity and the highest overall diagnostic accuracy. Albumin is a negative acute-phase reactant that decreases during systemic inflammation because of reduced hepatic synthesis and increased capillary leakage [17]. Combining ferritin, a marker of inflammation, with albumin, a marker of nutritional and physiological reserve, provides a composite measure that reflects disease severity more comprehensively.

In this prospective cohort of patients with septic shock, nonsurvivors had significantly higher ferritin-based biomarkers and severity markers than survivors. Although day 1 serum ferritin, Ferritin Index, FAR, SOFA, qSOFA, and lactate were associated with 28-day mortality on univariate analysis, SOFA remained the only independent predictor in multivariable analysis, indicating that the prognostic information provided by ferritin-based biomarkers may overlap with overall illness severity. The Ferritin Index demonstrated good discrimination (AUC 0.795), consistent with the potential prognostic role of ferritin-related markers reported in sepsis; however, Gunasekaran et al. found no association between ferritin/Ferritin Index and mortality in a prospective septic shock cohort [18].

FAR showed the highest numerical AUC in our study (0.849), although DeLong analysis demonstrated significant superiority only over Ferritin Index and not over day 1 ferritin or delta ferritin. This finding is directionally consistent with a large retrospective study reporting an association between FAR and 28-day mortality in sepsis, although its discriminatory performance was more modest [13].

These findings are consistent with previous studies reporting the prognostic utility of ferritin-based biomarkers in sepsis. Elevated serum ferritin has been associated with increased mortality [11], while disturbances in iron metabolism have been linked to adverse outcomes in critically ill septic patients [12]. More recently, Liu et al. demonstrated that the ferritin-albumin ratio is independently associated with 28-day mortality in patients with sepsis [13]. A comparison between the present study and previous literature is summarized in Table 11.

**Table 11:** Comparison of the present study with previous studies evaluating ferritin-based biomarkers

Study	Study Population	Biomarker Evaluated	Principal Findings
Fang et al., 2022 [11]	ICU patients with sepsis	Serum ferritin	Elevated ferritin was independently associated with increased mortality.
Brandtner et al., 2020 [12]	Critically ill patients with sepsis	Iron metabolism markers, including ferritin	Dysregulated iron metabolism and elevated ferritin were associated with adverse clinical outcomes.
Liu et al., 2023 [13]	Patients with sepsis	Ferritin-albumin ratio	Higher ferritin-albumin ratio independently predicted 28-day mortality.
Pierrakos and Vincent, 2010 [16]	Critically ill patients	Sepsis biomarkers	Composite biomarkers demonstrated superior prognostic performance compared with individual biomarkers.
Present study	Septic shock patients (n = 79)	Ferritin index, Day 1 Serum Ferritin, Delta Ferritin, Ferritin-Albumin ratio	Ferritin-albumin ratio demonstrated the highest predictive performance (AUC 0.849), followed by day 1 serum ferritin (AUC 0.840), ferritin index (AUC 0.795), and delta ferritin (AUC 0.737).

Overall, the present findings support the growing evidence that ferritin-derived biomarkers, particularly the ferritin-albumin ratio, provide valuable prognostic information in septic shock [19-21]. Because ferritin and albumin are routinely available laboratory parameters, these biomarkers may represent practical and cost-effective tools for early risk stratification in critically ill patients.

Limitations of the study

This study has several limitations. First, it was conducted at a single tertiary care center, which may limit the generalizability of the findings to other populations and healthcare settings. Second, the observational study design precludes the establishment of causal relationships between ferritin-based biomarkers and clinical outcomes. Third, the relatively small sample size may have reduced the statistical power to detect smaller effect sizes. Also, the small pilot sample resulted in substantial uncertainty around the estimated sensitivity and, consequently, the precision of the sample size estimate was limited. Fourth, delta ferritin analysis was restricted to patients who survived beyond ICU day 3 and had serial ferritin measurements available. Exclusion of patients who died earlier likely introduced survivor bias, as these patients probably had the greatest disease severity. Consequently, the reported diagnostic performance, particularly the high sensitivity of delta ferritin, may overestimate its performance in routine clinical practice. Accordingly, the utility of delta ferritin is limited to patients who survive until day 3 and undergo repeat ferritin measurement, restricting its applicability as an early prognostic marker in septic shock. Finally, the high mortality observed in this single-centre referral cohort may limit the external validity of the identified biomarker cutoff values. Because optimal prognostic thresholds are influenced by disease severity, patient characteristics, and healthcare setting, these cutoffs should be considered hypothesis-generating until validated in larger, multicenter studies involving more heterogeneous septic shock populations.

## Conclusions

Ferritin-based biomarkers demonstrated potential utility for risk stratification of 28-day mortality in patients with septic shock, with the ferritin-albumin ratio showing the highest discriminatory performance among the evaluated biomarkers. However, these biomarkers did not demonstrate independent prognostic value beyond established measures of illness severity, with SOFA remaining the only independent predictor of mortality in multivariable analysis.

Ferritin-based biomarkers may therefore serve as readily available adjunctive tools for early risk assessment rather than replacements for conventional severity scores. Given the single-center design, limited sample size, and potential survivor bias in the assessment of delta ferritin, these findings should be validated in larger, multicenter prospective cohorts before routine clinical implementation.
